# Modeling Diabetes Risk and Progression With Public Health Data: Ontology-Guided, Simulation-Capable Digital Twin Study

**DOI:** 10.2196/87374

**Published:** 2026-04-21

**Authors:** Qingrui Li, Kapileshwor Ray Amat, Eric L Johnson, Juan Li

**Affiliations:** 1 Department of Computer Science, North Dakota State University, PO Box 6050, NDSU Dept #2455, Fargo, ND, 58108, United States, 1 7012319662; 2 Department of Family and Community Medicine, School of Medicine & Health Sciences, University of North Dakota, Grand Forks, ND, United States

**Keywords:** digital twin, diabetes, public health datasets, Midlife in the United States, MIDUS, multiagent AI, ontologies, large language models, risk prediction, simulation, chronic disease

## Abstract

**Background:**

Digital twins (DTs) offer a paradigm for health care by enabling data-driven, simulation-capable representations of individual health trajectories. However, DT development remains limited by the scarcity of standardized, temporally structured, and multidomain data suitable for modeling chronic disease progression. Most existing DT studies rely on narrowly scoped or proprietary datasets, restricting generalizability. Public health datasets, such as the Midlife in the United States study, provide rich biopsychosocial information but are underused due to structural complexity and lack of semantic integration frameworks.

**Objective:**

This study aimed to develop and evaluate an ontology-guided, agent-orchestrated framework for constructing offline, simulation-capable, and progression-aware DTs from public health datasets. Using diabetes as a case study, the framework integrates agent-based orchestration, medical ontologies, and large language model (LLM)–assisted semantic reasoning with machine learning to support explainable feature structuring, risk prediction, and predictive “what-if” progression analysis.

**Methods:**

A 6-stage DT framework was developed and applied to Midlife in the United States wave 2 (baseline) and wave 3 (follow-up) data. Ontology- and LLM-assisted feature selection identified predictors across biological, behavioral, psychosocial, and socioeconomic domains. Cleaned and harmonized data were used to train predictive models (random forest, eXtreme gradient boosting, and logistic regression) to estimate diabetes onset at follow-up. A state-transition simulator was implemented to model between-wave progression dynamics, quantify transitions across low-, medium-, and high-risk states, and evaluate predictive “what-if” scenarios such as weight reduction and lifestyle improvement. Model performance was assessed using accuracy, *F*_1_ score, area under the receiver operating characteristic curve (AUC), and calibration metrics.

**Results:**

From 9976 candidate variables, ontology- and LLM-guided selection retained the top 200 relevant predictors spanning biological, behavioral, psychosocial, and socioeconomic domains. Predictive modeling achieved strong discrimination, with random forest (AUC=0.82, accuracy=0.76) and eXtreme gradient boosting (AUC=0.81, accuracy=0.75) outperforming logistic regression (AUC=0.78). The state-transition simulator reproduced realistic progression patterns: 33.9% (1414/4174) of participants changed risk states between waves, and the high-risk group increased from 10.8% (451/4174) to 32.2% (1344/4174). Next-state prediction accuracy reached 92.5%. Predictive “what-if” analyses showed that with a simulated 10% weight reduction, model-estimated diabetes cases decreased by 98 (from 576 to 478). A placebo test (0% weight change) produced less than 0.3% difference in risk distribution, confirming model stability.

**Conclusions:**

This study presents a foundational, ontology-guided, and agent-orchestrated framework for constructing offline, simulation-capable, and progression-aware DTs from public datasets. By combining semantic reasoning, multidomain predictors, and predictive “what-if” progression simulation, the framework transforms static population data into longitudinal, interpretable representations of individual health trajectories. The proof-of-concept application to diabetes demonstrates that public health data can support robust and explainable DT models for exploratory risk analysis and hypothesis generation, without implying causal intervention effects or direct clinical decision support.

## Introduction

### Background

Digital twins (DTs) are virtual representations of physical entities that are designed to represent and simulate real-world systems to predict and optimize outcomes [1]. In health care, DTs can serve as individualized, data-driven models of patients, enabling proactive monitoring, personalized intervention, and scenario-based simulation [2]. By integrating longitudinal biomedical, behavioral, psychosocial, and environmental data, DTs offer the potential to transform chronic disease management from reactive treatment to anticipatory care.

### Challenges in Health Care DTs

Despite this promise, the development of health care DTs is constrained by several challenges. First, there is a scarcity of high-quality, structured datasets that support temporal modeling and personalization, especially in chronic disease contexts [3]. Second, there is a lack of standardized frameworks for organizing heterogeneous health variables across domains and time points [4,5]. Finally, feature selection and semantic integration remain complex tasks, requiring both domain expertise and explainability [6]. These limitations are particularly acute for type 2 diabetes, where long-term behavioral, physiological, and psychosocial factors interact in complex, nonlinear ways. Current prototypes typically rely on narrowly scoped, proprietary datasets collected in controlled clinical settings, which limits generalizability and reuse and constrains their applicability beyond specific populations or case settings.

### Opportunity in Public Health Datasets

In contrast, large-scale public health datasets, such as the MIDUS (Midlife in the United States) study [7], the National Health and Nutrition Examination Survey [8], and the UK Biobank [9], contain rich biopsychosocial profiles of individuals over time. These resources offer an underused opportunity for constructing inclusive and population-grounded DT representations.

However, they present structural and semantic challenges, including inconsistent variable definitions, fragmented modules, missing values, and a lack of standardized feature hierarchies. To date, most uses of these datasets have focused on regression or correlation analyses rather than longitudinal, simulation-capable modeling of individual risk trajectories.

### Objective

This study aimed to develop a foundational, ontology-guided, and agent-orchestrated framework for constructing offline, simulation-capable DTs from public health datasets. In this work, we adopt a restricted and pragmatic definition of a health care DT, referring to an offline, simulation-capable virtual representation derived from population-level data, rather than a continuously synchronized or clinically validated dynamic twin.

Using diabetes risk prediction as a demonstrative case study, we sought to do the following:

1. Transform longitudinal public health data into temporally structured, semantically enriched feature sets suitable for DT modeling;
2. Integrate medical ontologies and large language model (LLM)–assisted semantic reasoning to support intelligent and explainable feature selection; and
3. Develop prediction and simulation models that support risk estimation and predictive “what-if” scenario analysis rather than causal intervention inference.

By addressing data reuse, semantic organization, and interpretable predictive simulation, this work establishes a new methodological pathway for constructing progression-aware and explainable DTs from population-level data, with a focus on predictive modeling and exploratory analysis rather than real-time operation, causal inference, or direct clinical decision-making.

## Methods

### Framework Design

#### Overview

**Figure 1.**
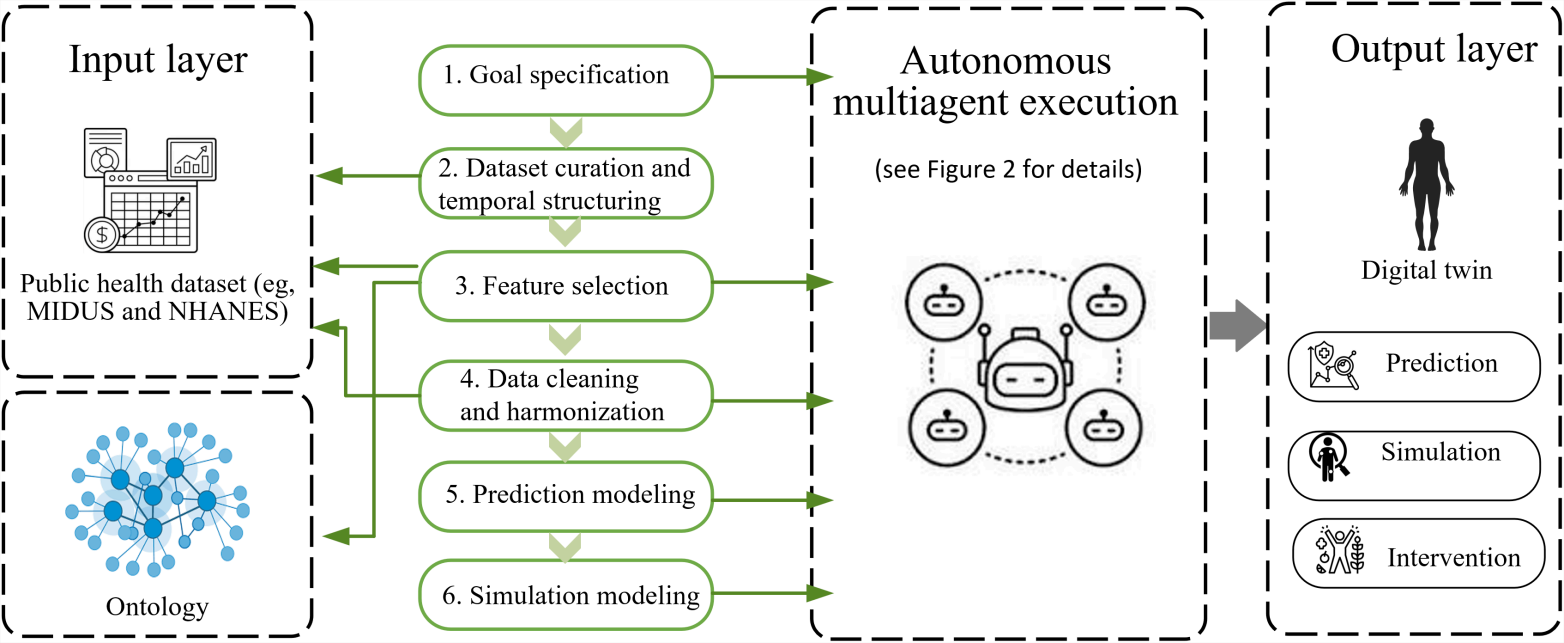
Multiagent, ontology-guided framework for constructing digital twins from public health data. MIDUS: Midlife in the United States; NHANES: National Health and Nutrition Examination Survey.

We developed a foundational, modular framework for constructing offline, simulation-capable DTs from public health data, illustrated using diabetes risk prediction in the MIDUS cohort. As illustrated in Figure 1, the framework is organized into six stages: (1) goal specification, (2) dataset curation and temporal structuring, (3) ontology- and LLM-assisted feature selection, (4) data cleaning and harmonization, (5) prediction modeling, and (6) simulation modeling.

These stages are coordinated through an agent-orchestrated architecture, in which specialized agents are assigned distinct roles, such as ontology mapping, LLM-based reasoning, data harmonization, prediction, and simulation. Agents operate as LLM-driven reasoning components with bounded autonomy, enabling semantic interpretation, ambiguity resolution, and iterative refinement rather than rigid rule-based execution.

This design supports scalability, transparency, and modularity, enabling each component to function independently while contributing to the overall DT framework without implying real-time synchronization, causal inference, or clinical decision-making capability.

#### Scope of the DT Implementation

The DT instantiated in this study is offline and population-trained, using paired observations from MIDUS waves 2 and 3. Models are trained once and applied deterministically during simulation. The system is intended for progression-aware research and hypothesis exploration, rather than real-time operation, causal inference, or clinical decision support.

#### Agent Autonomy and Interagent Reasoning

While the overall workflow of the framework follows a logical progression from data curation to prediction and simulation, the internal execution relies on agentic reasoning rather than rigid pipeline heuristics. Unlike traditional software modules that execute predefined rules (eg, “if X then Y”), the agents in this framework leverage LLMs to perform probabilistic reasoning, semantic interpretation, and adaptive decision-making.

**Figure 2.**
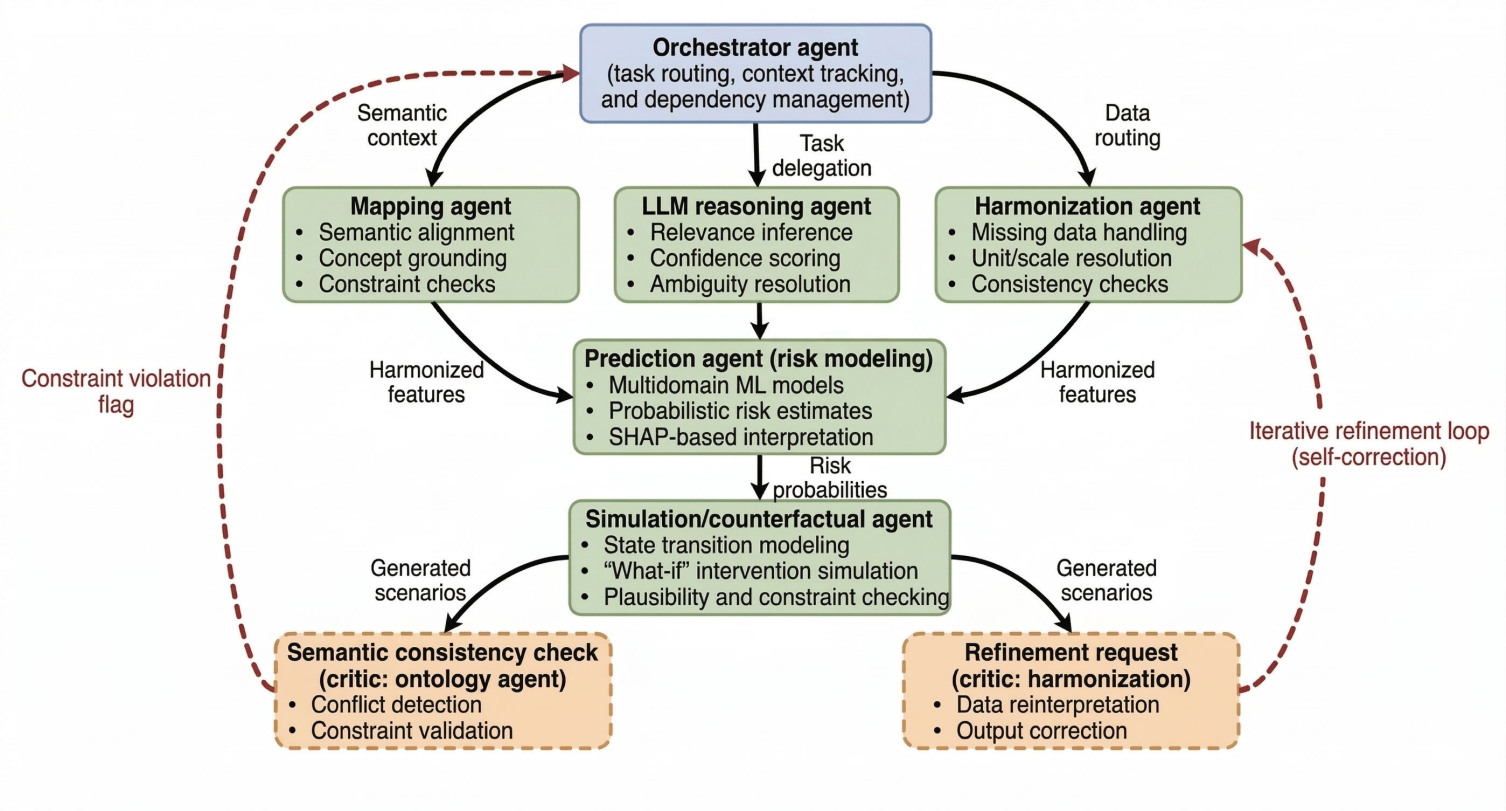
The multiagent collaboration architecture. Unlike a linear software pipeline, the orchestrator agent dynamically manages task execution based on semantic context. The architecture features explicit iterative refinement loops (red-dashed lines), where "Critic" functions (eg, semantic consistency check) autonomously flag errors or implausible simulations, triggering replanning and data reinterpretation by the functional agents. LLM: large language model; ML: machine learning; SHAP: Shapley Additive Explanations.

As illustrated in Figure 2, the framework departs from a static pipeline by employing a “Hub-and-Spoke” agentic topology centered on a dynamic orchestrator agent. This architecture enables 3 distinct forms of autonomy not present in the standard modular software:

1. Semantic adaptability: Agents resolve ambiguity in MIDUS variable definitions by inferring meaning from metadata, ontology context, and prior knowledge rather than requiring explicit hard-coded mappings.
2. Contextual interaction: Outputs produced by one agent condition the reasoning of downstream agents, and simulation outputs are checked against ontology constraints, enabling iterative refinement rather than one-pass execution.
3. Reflective evaluation: The simulation agent evaluates generated counterfactual scenarios for plausibility and internal consistency and regenerates outputs when conflicts are detected.

These behaviors distinguish the framework from a static processing pipeline and motivate the use of an agentic abstraction, while remaining intentionally lightweight and constrained compared to fully autonomous multiagent systems.

To complement the conceptual overview in Figure 2 and Table 1, we provide 2 brief execution traces (Textboxes1  2) illustrating (1) semantic ambiguity resolution during feature selection and (2) critic-triggered refinement during counterfactual simulation.

**Table 1.** Comparison between a traditional pipeline and the proposed agentic framework.

Aspect	Traditional pipeline	Proposed agentic framework
Logic	Deterministic rules	LLM^a^-based probabilistic reasoning
Ambiguity handling	Manual preprocessing	Semantic inference and confidence scoring
Interaction	Unidirectional	Iterative, context-conditioned refinement
Adaptation	None	Output regeneration under constraints
Failure mode	Error or crash	Reasoned approximation

a LLM: large language model.

Textbox 1. **Example trace: resolving semantic ambiguity in a MIDUS variable.**

Input (ambiguous item). The orchestrator agent receives the MIDUS item “Falling or staying sleep frequency - 30 days”, which conflates sleep-onset and sleep-maintenance difficulty and thus lacks a single unambiguous semantic label.

Ontology-grounded context. The orchestrator agent delegates grounding to the ontology mapping agent, which retrieves compact ontology evidence (top-matched concepts and a few relations) consistent with a broader sleep disturbance symptom frequency construct and packages it as a structured context for downstream inference.

Structured decision (LLM reasoning). Conditioned on the MIDUS metadata and retrieved ontology context, the LLM reasoning agent outputs a constrained JSON decision, eg,

{“classification”:“Indirect”,“relevance_score”:0.6,“reasoning”:“Sleep disturbance is associated with metabolic dysregulation and increased diabetes risk.”}

The orchestrator agent records a canonical label (eg, sleep_disturbance_symptom_frequency_30d) for harmonization and retains the feature in the ranked set used for modeling.

Reflective check (bounded loop). A Critic function validates schema/score range and checks semantic consistency with the retrieved evidence; if the score is below a predefined threshold, the orchestrator triggers a single re-query, otherwise the decision is accepted and logged.

Textbox 2. **Example trace: critic-flagged implausible counterfactual and orchestrated refinement.**

Input (candidate scenario). The simulation agent proposes a candidate “what-if” perturbation that attempts to vary a prescription-medicine checklist variable to reduce predicted diabetes risk; this proposal is subsequently screened by the Critic before any simulation is accepted.

Critic flag (implausible/nonactionable). A Critic function flags the scenario as nonactionable and semantically inconsistent with the DT's intervention scope: prescription history is a downstream proxy/outcome, not an upstream modifiable determinant for counterfactual exploration.

Orchestrator replanning (context-conditioned refinement). The orchestrator agent removes the nonmodifiable proxy variable and replans the scenario using upstream, modifiable factors already supported by the simulator (eg, body weight and physical activity). The simulator is rerun under these perturbations.

Refined output (accepted). The refined scenarios yield stable, monotonic risk changes consistent with the simulator’s sensitivity design (eg, 5% and 15% weight reductions decrease average predicted risk), and the accepted trace is logged.

### Goal Specification Using the Population, Intervention, Comparison, and Outcome Approach

Defining a clear objective is essential for aligning data engineering, modeling, and evaluation with actionable health care needs. In this framework, the objective is formalized through the Population, Intervention, Comparison, and Outcome (PICO) approach [10], which provides a structured foundation for prediction and simulation.

For this study, the following points should be noted:

- Population (P): adults aged ≥40 years in MIDUS wave 2, a group at elevated risk of type 2 diabetes due to age-related metabolic changes and cumulative lifestyle factors
- Intervention (I): a data-driven DT integrating multidomain predictors (eg, BMI, physical activity, sleep quality, psychosocial stress, and family history) derived from surveys and biomarkers to simulate diabetes risk
- Comparison (C): benchmark models using only biomedical or clinical variables, assessing the added value of including psychosocial and behavioral factors
- Outcome (O): diabetes onset at wave 3, defined through self-reported diagnosis, medication use, or biomarkers (fasting glucose and HbA_1c_)

This specification clarifies the DT’s purpose, guides variable selection, and defines evaluation benchmarks. The PICO structure also generalizes easily to other chronic diseases or datasets.

### Dataset Curation and Temporal Structuring

High-quality DTs require longitudinal data that capture changes in individual health states. Although MIDUS contains only 2 major waves, it provides sufficient longitudinal contrast for modeling transitions between baseline (wave 2) and follow-up (Wave 3).

The following procedures were applied:

1. Wave alignment and participant tracking: wave 2 served as baseline and wave 3 as follow-up. Participants with complete paired data were retained, yielding 4173 individuals with consistent identifiers and matched features.
2. Outcome labeling: diabetes onset was defined using wave 3 data (self-report, medication use, and biomarkers such as fasting glucose and HbA_1c_) to provide ground truth for supervised learning.
3. Feature harmonization: variables across biological, behavioral, psychosocial, and socioeconomic domains were standardized and harmonized. Comparable constructs (eg, BMI, activity, stress, and income) were aligned for consistency across waves.
4. Temporal pairing for transition modeling: instead of computing explicit Δ features, health progression was represented implicitly through paired records (wave 2 → wave 3). Each baseline profile served as model input and its follow-up state as the target.

This process transformed MIDUS from a general-purpose survey into a longitudinal, simulation-ready dataset consistent with DT principles. Although limited to 2 waves, the paired design enabled population-level transition modeling and individualized progression analysis over nearly a decade.

### Ontology-Assisted and LLM-Driven Feature Selection

#### Feature-Selection Overview

Public health datasets, such as MIDUS, include thousands of variables spanning biomedical, psychological, behavioral, and social domains. Selecting the most relevant features for DT construction requires scalable, explainable reasoning beyond traditional statistical filters. Conventional feature selection techniques, such as association tests [11], correlation thresholds [12], or heuristics [13], often overlook semantically subtle but influential variables (eg, stress or coping behavior).

To overcome this limitation, we developed a hybrid feature selection pipeline that integrates domain ontologies with LLM reasoning in a retrieval-augmented setting [14]. Feature selection is treated as a structured semantic reasoning task rather than a purely statistical filter [14].

#### Semantic Enrichment With Ontologies

Each MIDUS variable was mapped to a curated set of diabetes-related and study-design ontologies, enabling the LLM to reason across biomedical and psychosocial dimensions. The following ontologies were integrated:

- The Diabetes Mellitus Treatment Ontology (DMTO) [15] capturing treatment strategies, complications, and management protocols
- The Common Diabetes Medications Ontology [16] linking medication variables to pharmacological mechanisms (of action)
- The FHIR (Fast Healthcare Interoperability Resources) And SSN (Semantic Sensor Network)-based Type 1 diabetes Ontology (FASTO) [17] providing real-time monitoring and device-related concepts
- The BioMedBridges Diabetes Ontology [18] defining diagnostic and phenotypic concepts for translational research

Crucially, to contextualize the role of each variable within the MIDUS longitudinal design, we incorporated the Study Cohort Ontology [19,20]. This allowed the agent to understand the structural purpose of a variable (eg, as a baseline demographic, psychosocial instrument, or clinical outcome), which is essential for distinguishing direct from indirect relevance.

Variable metadata (labels, descriptions, and coding) were mapped using keyword matching, semantic similarity, and vector embeddings. The result was a structured, ontology-aligned representation that enabled the retrieval-augmented generation (RAG) agent to reason over deep conceptual relationships rather than surface-level text similarity.

In this framework, the ontology layer serves primarily as a semantic and organizational scaffold rather than as a direct performance-optimizing component. Its role is to (1) impose a consistent hierarchical structure over heterogeneous public health variables, (2) guide LLM-assisted feature selection toward domain-relevant predictors, and (3) enable interpretable grouping and reuse of features across modeling stages. Accordingly, ontology integration is intended to improve transparency, explainability, and reproducibility, rather than to guarantee higher predictive accuracy.

#### Relevance Classification With LLMs

After enrichment, the RAG agent evaluated each variable’s relevance to diabetes risk prediction using structured prompts containing ontology context and metadata. Variables were classified as directly relevant, indirectly relevant, or not relevant, each with a confidence score and explanatory note. The agent operated in an iterative loop: (1) extract variable metadata and ontology matches; (2) construct prompts integrating semantic context; (3) generate classification, justification, and confidence; (4) requery or reformulate if confidence is low; and (5) log outputs for transparency and reproducibility.

This process yielded a feature set annotated with ontology links, metadata, and reasoning traces, ensuring transparency, auditability, and explainability for downstream modeling.

The confidence scores generated by the LLM represent the internal consistency of the relevance classification given the ontology-grounded prompt context and were used to control iterative requerying rather than to impose a hard inclusion threshold. To assess face validity, we manually audited the resulting feature set with respect to established diabetes risk factors. Core predictors, such as age, BMI, blood pressure, family history, physical activity, smoking, mental health indicators, and medication use, were consistently retained, and we did not observe systematic exclusion of known clinical predictors. Disagreements primarily occurred for borderline psychosocial or survey-specific variables rather than for established biomedical risk factors.

The LLM-assisted feature selection is not intended to replace statistical feature selection or expert curation, nor is it strictly required to achieve high discrimination with flexible tree-based models. Its primary contribution lies in semantic coverage, explainability, and scalability when working with large, heterogeneous public health datasets, where manual curation is impractical and purely statistical filters risk omitting contextually relevant variables.

### Data Cleaning and Harmonization

Following feature selection, a standardized cleaning pipeline ensured data quality and consistency:

- Variable-type normalization: unified categorical, ordinal, and Boolean variables
- Missing-data handling: mean/mode imputation for low-missing features; k-nearest neighbor (kNN) imputation for complex patterns
- Scale and unit standardization: normalized continuous features and harmonized measurement units
- Semantic harmonization: merged equivalent constructs across waves via ontology tags
- Outlier detection: applied statistical thresholds and domain-specific plausibility rules

MIDUS contains both item-level missingness and wave-level attrition, which may correlate with health status and socioeconomic factors. We therefore do not assume that data are missing completely at random. We selected kNN imputation because it preserves multivariate relationships among mixed-type variables and avoids distributional assumptions required by parametric multiple imputation models, which are difficult to validate in high-dimensional survey data with thousands of heterogeneous variables. kNN uses observed similarity across individuals to infer plausible values and is widely used in biomedical machine-learning pipelines where predictive performance and internal consistency are the primary goals. Nevertheless, kNN does not correct for missing-not-at-random mechanisms, and any bias arising from differential dropout or nonresponse may propagate into downstream prediction and simulation.

### Prediction and Simulation Modeling

The final stage of the framework focuses on developing models that support both risk prediction and scenario-based simulation, which are 2 core capabilities of a DT.

#### Prediction Modeling

Prediction models estimate the likelihood of diabetes onset at follow-up using multidomain features. This component extends beyond traditional biomedical risk scores by incorporating behavioral, psychosocial, and socioeconomic predictors.

Model objectives emphasize personalization, interpretability, and temporal continuity, ensuring that predictions remain clinically meaningful and adaptable as health states evolve.

#### Simulation Modeling

Given the availability of only 2 MIDUS waves, the simulator implements a single-step, between-wave state-transition model (wave 2 → wave 3). Therefore, it supports coarse-grained progression modeling and scenario-based sensitivity analysis but not continuous, multistep temporal dynamics or online updating. The counterfactual simulations are predictive rather than causal: they quantify how model-estimated diabetes risk changes when selected inputs are perturbed, holding the learned model structure fixed. They do not represent real-world intervention effects. The simulation module operationalizes the DT’s capacity to model how an individual’s predicted diabetes risk evolves under changing behavioral and psychosocial conditions. Conceptually, the simulator is designed as a coarse-grained state-transition system, where each participant’s health state at a future timepoint is estimated as a function of their baseline characteristics and current states. These states summarize multidomain attributes, including biological (eg, BMI and hypertension), behavioral (eg, physical activity, smoking, and alcohol use), and psychosocial (eg, anxiety and depression), into interpretable clusters that represent distinct risk levels.

To model transitions, a supervised learning function approximates $P(S_{t+1}|S_{t},X_{t}),$ where $S_{t}$ and $S_{t+1}$ denote latent health states at consecutive waves and $X_{t}$ represents observed features. Given the availability of only 2 MIDUS waves, this formulation supports single-step, between-wave transition modeling rather than fine-grained or continuous temporal dynamics. This enables the simulator to reproduce observed between-wave progression patterns progression patterns and estimate next-state probabilities under hypothetical scenarios.

Counterfactual reasoning is implemented through predictive scenario simulation, in which modifiable factors (eg, body weight, physical activity, smoking, alcohol use, stress, and depression scores) are systematically perturbed to assess their influence on predicted transition probabilities and future diabetes risk. The simulator then recomputes the resulting state distributions and outcome probabilities using the trained transition and outcome models.

By comparing such simulated trajectories, the DT supports multifactor “what-if” analysis at both individual and population levels, enabling exploration of model sensitivity to changes in key risk factors. Methodologically, this simulation framework complements the predictive component: while prediction estimates a static risk at a given timepoint, the simulator enables exploratory analysis of coarse-grained health evolution across survey waves.

The counterfactual simulations implemented in this framework are predictive rather than causal. They quantify how model-estimated risk changes when selected input variables are perturbed and do not represent real-world intervention effects. Accordingly, these simulations should be interpreted as model-based sensitivity analyses and hypothesis-generation tools, rather than estimates of causal impact or decision-grade clinical recommendations.

#### Integration

Together, prediction and simulation form the methodological foundation of the DT. Prediction provides individualized risk estimates, whereas simulation allows for the exploration of dynamic scenarios and potential interventions. This dual design ensures that the DT framework not only forecasts outcomes but also offers actionable insights for prevention and management in diabetes and other chronic diseases.

### Agentic LLM Workflow and Reproducibility

The feature selection component is implemented as a retrieval-augmented, LLM-driven agent. We used OpenAI GPT-3.5 turbo (OpenAI Python SDK, openai>=1.0.0) with a temperature of 0.3 to reduce stochasticity. For each MIDUS variable, the agent receives the variable’s name and metadata together with ontology-grounded context retrieved from 6 diabetes-related ontologies: Diabetes Mellitus Diagnosis and Support Ontology, DMTO, BioMedBridges Diabetes Ontology, Common Diabetes Medications Ontology, FASTO, and the Study Cohort Ontology.

Additional details are provided here:

- Ontology RAG. Feature names and ontology concept labels are embedded using sentence transformers (allMiniLML6v2). For each feature, the topk =5 ontology concepts above a similarity threshold of 0.3 are retrieved. For each retrieved concept, ontology relationships are extracted as triples (eg, Type2Diabetes → hasRiskFactor → Obesity), and short concept explanations are generated and filtered. These labels, Internationalized Resource Identifiers, triples, and explanations form a structured context block injected into the LLM prompt.
- Prompt and output schema. The LLM is prompted with a fixed system instruction and a user template containing (1) MIDUS variable metadata, (2) retrieved ontology context, and (3) an explicit scoring rubric. The model must return valid JSON with fields {relevance_score ∈ [0,1], classification (Direct/Indirect/Unrelated), reasoning}.
- Iteration and thresholds. Features are processed in batches of 40. If a feature’s relevance score is below 0.3, it is requeried once with expanded ontology context; if it is still below the threshold, it is excluded. This bounded control flow ensures deterministic termination. Final selection is performed by ranking features by relevance_score and retaining the top 200.
- Reproducibility. We fix the feature-processing order, ontology-retrieval parameters, and LLM temperature and record all configuration parameters, data and ontology versions, and LLM outputs in run logs. Baseline selectors (L1-regularized logistic regression [LASSO] and MI) use random_state =42 to ensure deterministic comparisons.
- Agent orchestration layer. The orchestration layer acts as a central controller that sequentially invokes specialized agents, including ontology mapping, feature selection, data harmonization, prediction, and simulation. Agents do not communicate peer-to-peer; all interactions are mediated by the orchestrator. Feedback loops are implemented, wherein simulation outputs are checked for semantic consistency against ontology constraints and refined when inconsistencies are detected.

### Ethical Considerations

This study used publicly available, deidentified data and did not involve interaction with individuals or the use of identifiable private information; therefore, it did not involve a human subject as defined under 45 CFR 46.102 [21], and institutional review board review was not sought. Informed consent for this secondary analysis was not required. Data were accessed and analyzed in accordance with the applicable MIDUS and Inter-university Consortium for Political and Social Research data use terms [22]. To ensure compliance with these terms, the LLM was strictly provided with variable names, feature descriptions, and other documentation-level metadata; no participant-level or individual MIDUS data was entered into the LLM.

## Results

### Cohort Characteristics

After applying inclusion criteria, the analytic cohort consisted of 4174 participants with complete records in both MIDUS wave 2 (baseline) and wave 3 (follow-up), allowing individual trajectories to be tracked longitudinally. The sample size, therefore, remained constant across waves (n=4174), although the number of participants with diabetes increased over time. The mean age at baseline was 56.0 (SD 12.4) years; 54.8% (n=2288) were female. By wave 3615 (14.7%), participants met the diabetes definition, indicating a moderate class imbalance.

As shown in Table 2, across both waves, participants who had or developed diabetes were older, more often male, had lower education and household income, and showed higher cardiometabolic burden (eg, BMI and hypertension) than those without diabetes. Behavioral differences were smaller, but people with diabetes reported slightly higher levels of light physical activity at follow-up, possibly reflecting postdiagnosis behavior changes. By wave 3, depressive symptoms were also higher in the diabetes group.

**Table 2.** Descriptive statistics of the study cohort (MIDUS waves 2 and 3), stratified by diabetes status.

Variable	MIDUS^a^ 2 overall (n=4174)	No diabetes (n=3730)	Diabetes (n=444)	*P* value	MIDUS 3 overall (n=4174)	No diabetes (n=3559)	Diabetes (n=615)	*P* value
Demographics								
Age (y), mean (SD)	56.0 (12.4)	55.4 (12.3)	61.6 (11.2)	<.001	64.0 (11.4)	63.3 (11.4)	68.5 (10.7)	<.001
Male, n (%)	1886 (45.2)	1643 (44.0)	243 (54.7)	<.001	1886 (45.2)	1561 (43.9)	325 (52.8)	.004
Female, n (%)	2288 (54.8)	2087 (56.0)	201 (45.3)	<.001	2288 (54.8)	1998 (56.1)	290 (47.2)	.004
Education (y), mean (SD)	7.3 (2.5)	7.3 (2.5)	6.6 (2.6)	<.001	7.6 (2.5)	7.7 (2.5)	7.0 (2.6)	<.001
Household income, median (IQR), $	57,500 (29,000-95,272)	59,500 (30,250-97,500)	46,250 (22,000-75,024)	<.001	68,875 (34,250-119,938)	72,500 (36,938-123,750)	55,250 (25,000-95,250)	<.001
Biological factors								
BMI, mean (SD)	27.9 (5.7)	27.4 (5.4)	31.9 (6.9)	<.001	28.2 (6.1)	27.6 (5.6)	31.5 (7.6)	<.001
Hypertension, n (%)	1263 (30.3)	982 (26.3)	281 (63.3)	<.001	1049 (38.9)	786 (34.6)	263 (61.6)	<.001
Behavioral factors								
Ever smoked, n (%)	2022 (65.1)	1782 (64.5)	240 (69.4)	.09	1321 (62.4)	1118 (62.1)	203 (64.2)	.51
Light activity (h/wk), mean (SD)	2.0 (1.5)	2.0 (1.5)	2.3 (1.7)	<.001	2.0 (1.5)	1.9 (1.5)	2.5 (1.7)	<.001
Psychosocial factors								
Depression score, mean (SD)	0.6 (1.7)	0.6 (1.7)	0.6 (1.7)	.91	0.6 (1.7)	0.5 (1.6)	0.7 (1.9)	.03

a MIDUS: Midlife in the United States.

### Feature-Selection Outcomes

Ontology- and LLM-assisted feature selection yielded a curated set of predictors spanning biological, behavioral, psychosocial, and socioeconomic domains. A representative subset is shown in Table 3. As expected, classic biomedical risk factors—BMI, hypertension, waist-to-hip ratio, and current weight—were classified as directly relevant. The pipeline also surfaced function- and lifestyle-related variables (eg, basic activities of daily living, shortness of breath while walking, and health limits walking >1 mile), which capture mobility and cardiopulmonary reserve and are not always selected by purely statistical filters. In addition, socioeconomic (household income) and psychosocial (anxiety symptoms) variables were retained as indirectly relevant, extending the model beyond a biomedical-only view of diabetes risk.

**Table 3.** Representative variables selected by ontology- and LLM^a^-assisted feature selection for diabetes risk prediction.

Variable	Domain	Relevance classification	Ontology alignment (examples)	Notes/explanation
Body Mass Index - Metric	Biological	0.8 (direct)	DMTO^b^: 0000362 (overweight/obesity)	BMI is a well-established indicator of obesity and a major risk factor for diabetes.
Age	Biological	0.8 (direct)	DMDSONT^c^: Patient_hasAge; SNOMED CT^d^: chronological age	Age is a strong predictor due to accumulated lifestyle exposures and metabolic changes.
Hypertension (ever diagnosed, 12 mo)	Biological	0.8 (direct)	DMDSONT: Hypertension_b	Hypertension is a known comorbidity and a direct risk factor for type 2 diabetes.
Any chronic conditions (12 mo)	Biological	0.8 (indirect)	DMTO: DDO_0000201 (chronic disease)	Multimorbidity contributes to systemic inflammation and increases diabetes risk.
Weight compared to 5 years ago	Biological	0.8 (indirect)	DMTO: DDO_0000130 (weight trajectory)	Weight gain over time is closely associated with higher diabetes risk.
Current weight (pounds)	Biological	0.8 (direct)	DMTO: DDO_0000130 (weight)	Absolute weight is a central predictor of diabetes risk.
Waist-to-hip ratio	Biological	0.8 (direct)	DMTO: DDO_0000320 (waist circumference)	Central obesity predicts diabetes more strongly than BMI alone.
Basic activities of daily living (3-item)	Behavioral	0.7 (indirect)	DMTO: DDO_0000127 (activity level)	Functional limitations are correlated with higher chronic disease risk.
Shortness of breath while walking	Behavioral	0.7 (indirect)	DMTO: DDO_0009259 (respiratory function)	Reduced capacity reflects poorer cardiometabolic health.
Health limits walking >1 mile	Behavioral	0.7 (indirect)	DMTO: DMTO_0001830 (walk)	Mobility restrictions often signal underlying cardiometabolic burden.
Household income	Socioeconomic	0.6 (indirect)	FASTO^e^: socioeconomic factor	Lower income is associated with limited health care access and higher diabetes risk.
Anxiety symptoms	Psychosocial	0.6 (indirect)	DMTO: DDO_0000483 (anxiety)	Anxiety can indirectly influence diabetes risk via stress responses and lifestyle.

a LLM: large language model.

b DMTO: Diabetes Mellitus Treatment Ontology.

c DMDSONT: Diabetes Mellitus Diagnosis and Support Ontology.

d SNOMED_CT: Systematized Nomenclature of Medicine—Clinical Terms.

e FASTO: FHIR (Fast Healthcare Interoperability Resources) And SSN (Semantic Sensor Network)-based Type 1 diabetes Ontology.

To support transparency, the full ranked list of the top 200 features, including ontology alignment, relevance level, and notes, is provided in Multimedia Appendix 1. These results confirm that the ontology-guided, multiagent pipeline can identify multidomain predictors consistent with a biopsychosocial model of diabetes.

To evaluate the specific contribution of the ontology-guided RAG agent, we conducted a comparative ablation study against 2 established statistical baselines:

1. Lasso: a standard method for sparse feature selection that penalizes nonzero coefficients.
2. Mutual information (MI): a univariate filter that selects features based on their statistical dependency with the target.

All methods were restricted to a top-K setting (K=200) to ensure a fair comparison of information density. The complete list of 200 features can be found in Multimedia Appendices 1-3. Selection quality was evaluated based on downstream predictive performance (area under the receiver operating characteristic curve [AUC]) and a manual semantic audit of feature relevance.

As shown in Table 4, the ontology-guided RAG agent achieved an AUC of 0.82, outperforming Lasso (AUC=0.73) and MI (AUC=0.78). This indicates that the agent identifies predictive signals that purely statistical filters overlook.

**Table 4.** Ablation study—feature-selection method comparison.

Feature-selection method	Number of features selected	AUC^a^ (prediction)	“Noise” features included^b^	Clinical relevance score^c^
Baseline 1: mutual information	200	0.78	Medium (110)	Low
Baseline 2: Lasso^d^ (L1)	200	0.73	High (135)	Medium
Ours (ontology+LLM^e^ agent)	200	0.82	Low (70)	High

a AUC: area under the receiver operating characteristic curve.

b “Noise” features refer to variables classified through manual semantic audit as artifacts or administratively correlated variables without clear causal or actionable relevance to diabetes risk.

c Clinical relevance score indicates the overall qualitative relevance of the selected feature set to established diabetes risk factors, based on manual semantic audit.

d Lasso: L1-regularized logistic regression.

e LLM: large language model.

**Figure 3.**
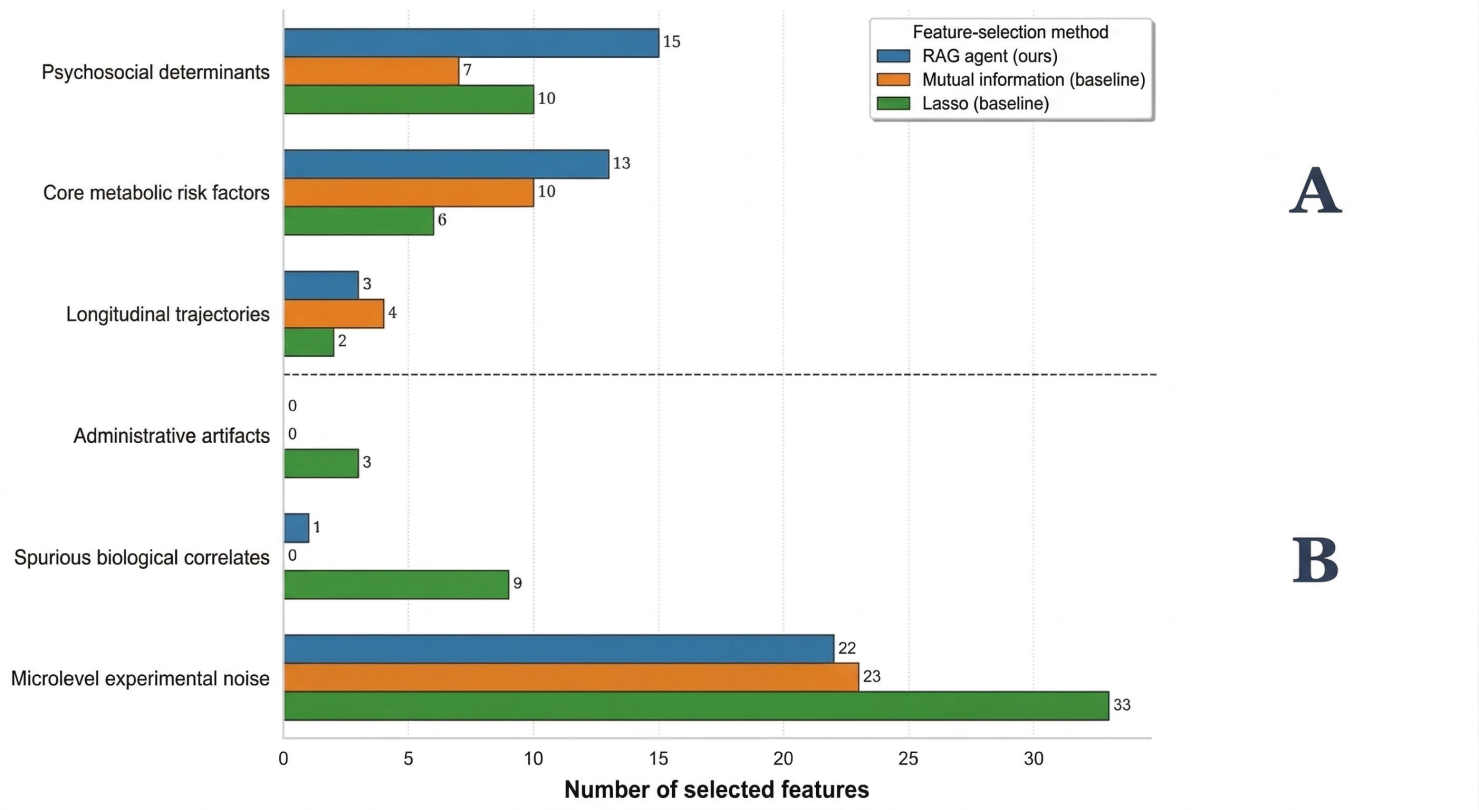
Comparative semantic audit of feature selection. (A) Target-relevant features (higher count is better) and (B) artifactual and noisy features (lower count is better). The RAG agent (blue) selects significantly more target-relevant features (eg, psychosocial determinants and metabolic risk factors) while suppressing artifactual noise compared to L1-regularized logistic regression (Lasso, green) and mutual information (orange). RAG: retrieval-augmented generation.

Beyond raw accuracy, the agentic approach demonstrated superior semantic precision. Statistical methods frequently selected “noisy” artifacts: variables that correlate with the target but lack causal validity.

- Reduction of artifacts: Lasso selected 135 features classified as noise or administrative artifacts (eg, “Interviewer ID” and “Data Collection Site”), whereas the RAG agent reduced this to 70, effectively filtering out spurious correlations.
- Actionable versus proxy features: as detailed in Figure 3, the RAG agent prioritized modifiable lifestyle constructs essential for DT simulation, selecting 21 physical activity items compared to only 4 for Lasso. Conversely, Lasso heavily relied on “Prescription Medicine” checklists (31 items vs 7 for RAG). While prescription history is a strong statistical proxy for disease, it represents a downstream outcome rather than an upstream, modifiable risk factor. By favoring upstream determinants (eg, “Lose 10 pounds by diet”), the agentic framework ensures the resulting DT is actionable.

Figure S1 and Tables S1–S5 (Multimedia Appendix 4) provide a detailed ablation and semantic analysis of feature-selection methods. These results show that ontology-guided RAG selects a substantially different and nonredundant feature set relative to Lasso and MI, with greater emphasis on modifiable lifestyle and psychosocial constructs and fewer spurious or administrative artifacts, thereby supporting its suitability for interpretable, intervention-oriented DT modeling.

### Prediction Performance

Prediction models were trained on wave 2 features to predict diabetes onset in wave 3. Of the 4174 participants, 615 (14.7%) developed diabetes, so the synthetic minority oversampling technique was applied during training to mitigate class imbalance. Three models were evaluated: random forest, eXtreme gradient boosting, and logistic regression. A 5-fold cross-validation with stratified splits was used.

All models showed good discrimination, with tree-based models performing the best:

- Random forest: accuracy 0.856, AUC 0.826
- eXtreme gradient boosting: accuracy 0.868, AUC 0.829
- Logistic regression: accuracy 0.807, AUC 0.81

Performance improved steadily as additional domains were added. Models restricted to biological variables reached only about AUC ≈ 0.75; adding behavioral variables increased performance to about AUC ≈ 0.79; and using the full multidomain set (biological + behavioral + psychosocial + socioeconomic) yielded the best performance (AUC up to 0.82). This pattern underscores that psychosocial and functional factors in MIDUS carry incremental signals for diabetes onset.

**Figure 4.**
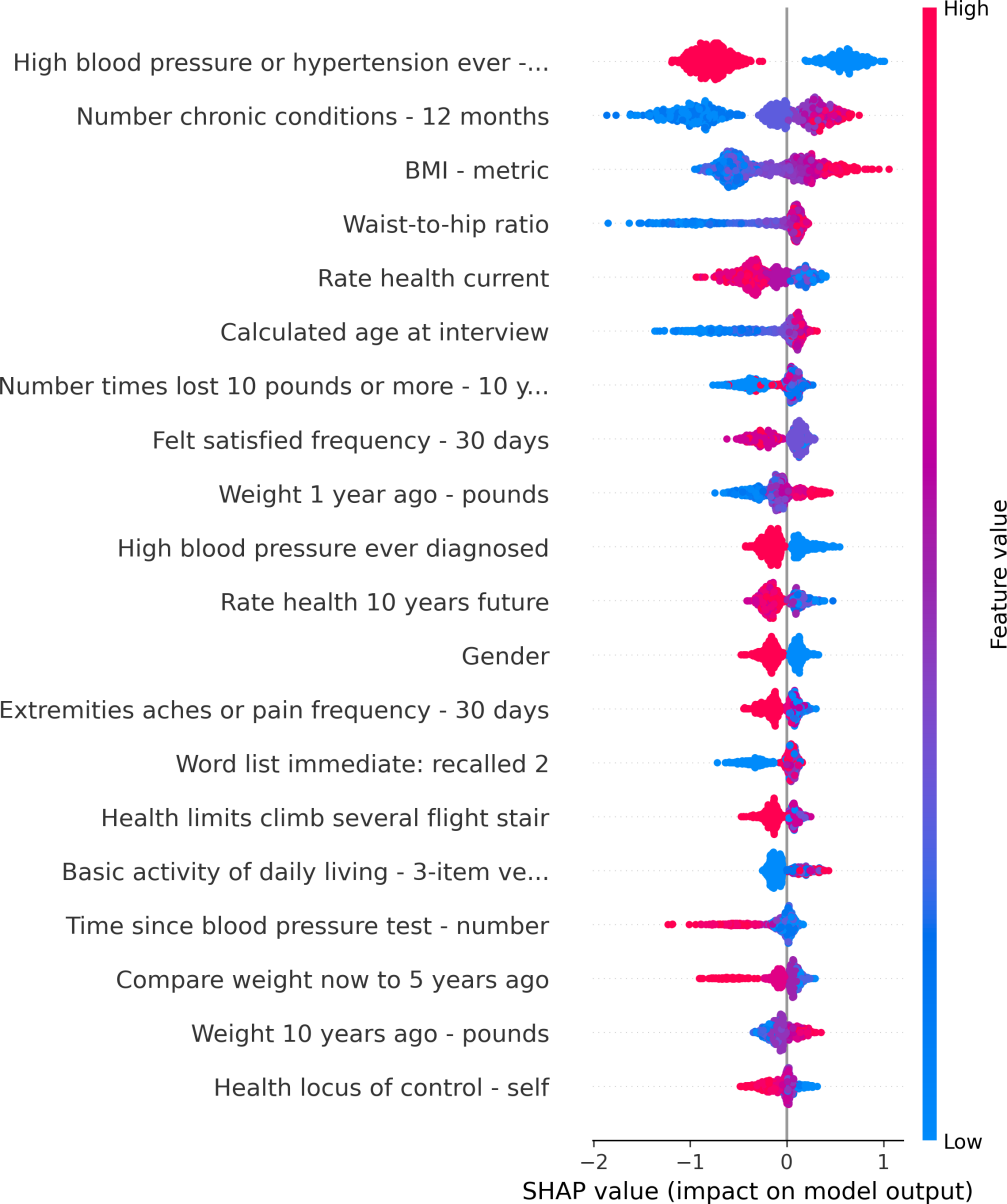
SHAP summary of top 20 features. SHAP: Shapley Additive Explanations.

Model interpretability was examined with the SHAP (Shapley Additive Explanations) analysis [23]. As shown in Figure 4, the highest impact features included hypertension status, number of chronic conditions, BMI, waist-to-hip ratio, several functional-limitation items, and indicators of health monitoring (eg, time since the last blood pressure test). Together, these suggest that the learned model is capturing not only metabolic risk but also the accumulation of chronic disease burden and health care–engagement patterns.

### Simulation Findings

#### State Construction and Risk Stratification

Discrete risk states were introduced to support interpretable, coarse-grained transition modeling, given the sparse temporal structure of the data. Clustering is used here only as a discretization of a continuous predicted-risk score into interpretable strata. We selected K=3 to align with standard clinical stratification into low-, medium-, and high-risk categories. When K>3, the clustering yields finer-grained strata of the same risk distribution rather than a different disease model. To preserve semantic comparability across K, we define the high-risk population as the union of the highest-risk clusters whose combined size matches the K=3 high-risk group.

Alternative state definitions could be used, including quantile-based binning of predicted risk, latent class or hidden Markov models, or continuous-state formulations that avoid discretization altogether. We selected k-means for this study because it provides a transparent and data-driven partition of the risk distribution that is compatible with sparse 2-wave data. Exploring continuous-time and latent-state models is an important direction for future work as higher-frequency data become available.

We fixed the centroids learned at wave 2 and applied them to wave 3, so that states were compared between waves.

- Wave 2: low 79.1% with mean risk 3.03% (SD 3.35%), medium 10.1% with mean risk 27.8% (SD 10.77%), and high 10.8% with mean risk 93.5% (SD 10.84%)
- Wave 3: low 62.4% with mean risk 1.20% (SD 5.84%), medium 5.4% with mean risk 49.7% (SD 7.75%), and high 32.2% with mean risk 90.8% (SD 9.77%)
- Participants changing states: 1414/4174 (33.9%)

The substantial increase in the high-risk group (10.8% → 32.2%) and one-third of participants who changed states indicate meaningful between-wave shifts in modeled diabetes risk, supporting the use of a transition-based, coarse-grained simulator.

#### Transition Model (Wave 2 → Wave 3)

We trained a state-transition model to estimate

$P(S_{W3}|S_{W2},x_{W2})$ where $S_{W2}$ and $S_{W3}$ represent cluster-based risk states and $x_{W2}$ is the participant’s wave 2 feature vector. Given the availability of only 2 time points, this model captures single-step, between-wave transitions rather than continuous temporal dynamics. Five-fold cross-validation with stratified splits was used. Inputs included demographics, biological, behavioral, psychosocial, and socioeconomic features. State-transition model performance is summarized in Table 5.

**Table 5.** State-transition model performance.

Model	Accuracy (SD), %	Macro *F*_1_, %	Average log-likelihood
XGBoost^a^	92.54 (1.39)	91.64	–0.2572
Random forest	88.57 (1.31)	79.42	–0.4089
Logistic regression	60.42 (2.31)	50.22	–0.9475

a XGBoost: eXtreme gradient boosting.

Tree-based models substantially outperformed the linear baseline, capturing nonlinear interactions among M2 features and baseline states. Model calibration was further assessed with Brier scores (≈0.07) and expected calibration error (≈0.04), indicating well-calibrated predictive transition probabilities.

#### Outcome Model (Diabetes at Wave 3)

To link state evolution to clinical outcomes, we trained a second model to estimate

$P\left(Y_{W3}=1|S_{W2},S_{W3},x_{W2}\right)$, where $Y_{W3}$ denotes diabetes onset at follow-up. Inputs included baseline features, wave 2 state probabilities, and predicted wave 3 state probabilities (soft outputs) from the transition model.

All 3 models achieved strong discrimination; tree-based models again delivered slightly better calibration, as shown in Table 6. This 2-stage design (state transition → outcome) supports progression-aware predictive modeling, consistent with the framework’s DT abstraction.

**Table 6.** Diabetes outcome model performance.

Model	AUC^a^ (SD), %	PR-AUC^b^, %	Brier score
Random forest	90.89 (2.90)	75.60	0.0640
XGBoost^c^	90.34 (2.80)	75.10	0.0638
Logistic regression	88.69 (1.91)	67.45	0.1089

a AUC: area under the receiver operating characteristic curve.

b PR-AUC: precision-recall area under the curve.

c XGBoost: eXtreme gradient boosting.

#### Predictive “What-If” Simulator

We then used the trained transition and outcome models as a predictive scenario-analysis engine. Modifiable wave 2 covariates (weight/BMI, physical activity, smoking, alcohol consumption, anxiety, and depression) were perturbed, and the models were rerun to obtain new wave 3 state distributions and predicted diabetes risk predictions. All simulation runs were deterministic and logged for reproducibility.

#### Scenario 1: 10% Weight Reduction (Predictive Sensitivity Analysis)

A uniform 10% decrease in weight (and hence BMI, assuming a fixed height) was applied to all 4174 participants (Table 7).

- Participants with any state change: 280/4174 (6.7%)
- Largest single improvement: 53 transitions from high → low risk
- The model-estimated number of diabetes cases decreased from 576 to 478 (−98) under this perturbation
- Individuals with reduced predicted risk: 2417/4174 (57.9%)

These changes reflect the model’s sensitivity to weight-related inputs rather than the projected real-world intervention effects.

**Table 7.** Effect of 10% weight/BMI reduction (predictive simulation).

Outcome	Preintervention	Postintervention	Change
Predicted diabetic (count)	576	478	–98
Any state change, n (%)	—^a^	280 (6.7)	—
High → low-risk transitions	—	53	—
Individuals with reduced risk, n (%)	—	2417 (57.9)	—

a Not applicable.

#### Scenario 2: Composite Lifestyle Scenario (Predictive Sensitivity Analysis)

A composite lifestyle scenario was simulated by increasing physical activity by 1 ordinal category (≈20% of the MIDUS 1‐6 scale), switching current smokers to nonsmokers, reducing alcohol consumption by 2 units, and reducing anxiety and depression scores by 20%.

Model-estimated response includes the following:

- Participants with state change: 300/4174 (7.2%)
- High → low improvement: 49 participants
- The model-estimated number of diabetes cases decreased from 576 to 506 (−70)
- Individuals with lower diabetes risk: 1417/4174 (33.9%)

Because MIDUS stores activity on an ordinal scale rather than as minutes per week, the “20% increase” was operationalized as a +1 shift in the activity category, which preserves the semantic meaning of the item.

#### Illustrative Case Studies

To demonstrate individual-level exploratory use, we simulated 2 patients:

- Case 1 (10% weight loss): 50-year-old male, BMI 29.6 kg/m², baseline predicted diabetes risk 35.4%. After perturbing weight-related inputs to reflect a 10% reduction (BMI 26.6 kg/m²), the model-estimated risk decreased to 22.1% (−13.3% points).
- Case 2 (composite lifestyle scenario): A 57-year-old male with a baseline risk of 57.6%. After increased activity, smoking cessation, reduced alcohol intake, and a 20% reduction in lower anxiety/depression scores, the predicted risk dropped to 36.0% (−21.4% points).

These vignettes show that the same population-trained simulator can support individualized, predictive “what-if” reasoning, rather than personalized intervention recommendations.

#### Sensitivity and Robustness Checks

To evaluate the stability of the proposed framework and the robustness of the latent state definitions, we conducted a series of sensitivity analyses examining alternative perturbation magnitudes, state-definition choices, placebo perturbation, and subgroup calibration:

- Alternative perturbation magnitudes: Weight reductions of 5% and 15% yielded monotonic decreases in average predicted diabetes risk (−6% and −14%, respectively).
- State-definition robustness: For K>3, the high-risk state was defined as the union of the highest risk clusters whose combined size matched the K=3 high-risk group. Under this definition, the composition and clinical profile of the high-risk population remained stable for K=3‐5 (>88% Jaccard overlap; Table S7 in Multimedia Appendix 4), and transition accuracy varied by <2%.
- Placebo test: A 0% weight change produced <0.3% difference in risk distribution, confirming model stability.
- Subgroup calibration: AUCs were consistent across age, sex, and income tertiles (variation <3%), indicating no major bias within the modeled population.

To assess the robustness of the latent state definitions, we performed a sensitivity analysis by varying K from 3 to 5. The “High Risk” state demonstrated high stability, maintaining a Jaccard membership overlap of >0.90 across configurations (see Table S7 in Multimedia Appendix 4). Increasing K primarily resulted in the granular subdivision of the “Healthy” state (eg, distinguishing normative vs optimal profiles) rather than altering the boundaries of the pathological state, confirming that the diabetic phenotype represents a distinct manifold in the data.

## Discussion

### Principal Results

This study demonstrates the feasibility of constructing a simulation-capable DT from a public, survey-based dataset using MIDUS waves 2 and 3. By integrating multidomain predictors, ontology-guided semantic structuring, and a 2-stage prediction–simulation design, the framework achieved strong diabetes risk prediction and reproduced meaningful between-wave risk transitions.

Our results highlight a critical limitation of traditional statistical feature selection in health informatics: the inability to distinguish causality from correlation. Although statistically rigorous, Lasso identified “Prescription Medicine” checklists as top predictors. In a static risk model, these are useful proxies; however, for a DT simulation, they are dead ends: one cannot “simulate” removing a history of hernia repair to cure diabetes. In contrast, the agentic framework successfully retrieved upstream determinants, such as dietary behavior and physical activity frequency. This shift from proxy-based prediction toward modifiable-input simulation justifies the computational complexity of the LLM layer, as it enables structured predictive sensitivity analysis (eg, “What if the patient increases vigorous activity?”) that statistical baselines cannot support. Across scenarios, observed changes should be interpreted as responses of the trained model to input perturbations rather than estimates of real-world intervention effects.

### Generalizability and Portability to Other Diseases and Datasets

Although diabetes is used as a case study, the proposed framework is modular and largely reusable across chronic conditions and public-health datasets. Disease-agnostic components include (1) the data harmonization and temporal alignment pipeline, (2) the agent-orchestration and logging layer (bounded autonomy, traceable message passing), (3) the general ontology-RAG retrieval mechanism (embedding-based concept retrieval + structured context injection), (4) the downstream prediction stack (tree-based and linear models with calibration/SHAP), and (5) the sensitivity-analysis interface that perturbs modifiable inputs and re-evaluates model outputs. Disease-specific components primarily include (1) the ontology set used for grounding retrieval, (2) the prompt rubric and relevance criteria used by the feature-selection agent (what constitutes “direct/indirect relevance” for a target disease), (3) the outcome definition (case ascertainment and temporal window), and (4) the selection of modifiable factors emphasized in the scenario analysis.

Porting the framework to a different chronic disease (eg, cardiovascular disease [CVD] or chronic obstructive pulmonary disease [COPD]) would involve four practical steps: (1) define the target end point and prediction horizon consistent with the dataset (eg, incident CVD event, COPD exacerbation, and hospitalization), (2) swap or extend the ontology layer using disease-appropriate ontologies/terminologies (eg, condition-specific risk-factor and phenotype ontologies, and mappings to the Systematized Nomenclature of Medicine [SNOMED] and International Classification of Diseases [ICD], where applicable), (3) update the agent prompt templates and scoring rubric so that relevance classification and confidence scoring reflect domain-specific pathways (eg, lipids/blood pressure for CVD; smoking history, spirometry proxies, and symptom burden for COPD), and (4) retune the state definition and progression model to match disease dynamics and data cadence. In datasets with sparse waves (as in MIDUS), coarse-grained discretization and single-step transitions can provide an interpretable progression abstraction; however, for conditions with faster dynamics or richer longitudinal sampling, the same pipeline could replace k-means discretization with quantile bins or latent-state models (eg, hidden Markov models) and replace between-wave transitions with continuous-time or event-based progression models.

Finally, we note that portability also depends on dataset structure. Survey-based cohorts provide multidomain exposures but limited clinical end points, whereas electronic health record (EHR)–based cohorts provide richer outcomes but noisier measurement and coding artifacts. The framework’s ontology-grounded RAG and semantic auditing steps are intended to mitigate such heterogeneity by making feature selection and simulation inputs more interpretable and traceable, while still requiring disease- and dataset-specific end point definitions and validation protocols.

### Limitations

#### Data Quality, Missingness, and Measurement Error

This study relies on the MIDUS dataset, which consists largely of self-reported behavioral, psychosocial, and health measures. Such data are subject to recall bias, social desirability bias, and reporting inaccuracies, particularly for diet, physical activity, mental health, and substance use. These errors may attenuate or distort learned associations and propagate uncertainty into both risk predictions and counterfactual simulations. Although large sample sizes and robust machine-learning models can mitigate random noise, systematic reporting bias remains an inherent limitation of survey-based DT construction.

MIDUS also exhibits item nonresponse and wave-level attrition that are likely to be partially missing not at random. For example, participants with poorer health may be less likely to complete follow-up surveys, and sensitive behaviors may be systematically underreported. While kNN preserves multivariate structure and reduces information loss, it cannot recover information that is systematically missing. Consequently, both predicted risks and simulated outcomes may be biased if missingness is correlated with unobserved health status. Future work should explore explicit missingness indicators, sensitivity analyses, or joint models of outcome and dropout when higher frequency or linked EHR data become available.

#### Limited Temporal Resolution

The framework is trained on only 2 observation points (MIDUS waves 2 and 3), separated by approximately a decade. This supports single-step, between-wave transition modeling but does not capture short- or medium-term disease dynamics, nor does it allow continuous updating or multistep forward simulation. As a result, the proposed DT should be viewed as a coarse-grained, long horizon progression model rather than a high-resolution temporal simulator. Additional waves or higher-frequency data (eg, EHRs or wearables) would be required to support continuous or multistep DT updating.

#### Predictive (Not Causal) Nature of Counterfactuals

The counterfactual “what-if” simulations implemented in this framework are predictive rather than causal. They assume that the statistical associations learned by the transition and outcome models remain stable under hypothetical interventions (eg, weight loss, increased physical activity, and smoking cessation). Accordingly, quantities such as “98 fewer diabetes cases” represent model-estimated or projected changes in risk under perturbed inputs, not real-world treatment effects.

While placebo and monotonicity tests demonstrate internal numerical stability of the simulator, they do not establish causal efficacy. Therefore, the simulations reflect model sensitivity rather than physiologically or clinically validated intervention outcomes and should be interpreted as sensitivity analyses and hypothesis generation tools rather than the estimates of clinical impact. Although the magnitude and direction of predicted effects are broadly consistent with major lifestyle intervention trials (eg, the Diabetes Prevention Program), such comparisons are illustrative only and do not constitute external validation or causal inference.

#### Clinical Applicability

The current framework is not intended for direct clinical deployment. Outputs such as transitions between cluster-based risk states or changes in population-level predicted incidence are not directly actionable at the individual patient level. Instead, the system is designed to support exploratory analysis, hypothesis generation, and population-level planning rather than point-of-care decision-making. Substantial additional work would be required for clinical translation, including calibration to clinical end points, alignment with guideline-based thresholds, and integration with EHR workflows.

Model-based simulations of hypothetical lifestyle changes also raise ethical and communication concerns. Without causal validation, simulated risk reductions could be misinterpreted as guaranteed outcomes, potentially leading to false reassurance or undue anxiety. Any future clinical deployment would require careful attention to explainability, uncertainty communication, and clinician oversight.

#### State Definition and Discretization

Health states were defined using k-means clustering for its simplicity, transparency, and compatibility with sparse 2-wave data. However, alternative discretization strategies—such as quantile-based risk bins, latent class models, hidden Markov models, or continuous-state formulations—could be used to define health states without hard clustering. Exploring such alternatives, particularly in datasets with higher temporal resolution, represents an important direction for future work.

#### Scope of the DT

The proposed framework does not implement several features of mature health care DTs, including continuous synchronization with real-world data, online learning, bidirectional patient feedback, or causal intervention modeling. It should therefore be interpreted as a foundational, offline, simulation-capable DT suitable for population-level exploration and hypothesis generation rather than a clinically deployed or continuously updating patient-specific twin.

### Comparison With Prior Work

Most existing health care DT prototypes have relied on narrowly scoped, proprietary datasets, often collected in clinical settings with limited generalizability. Prior studies in diabetes modeling [24-26] have focused primarily on short-term glucose forecasting or clinical risk scoring, with relatively little emphasis on integrating psychosocial and behavioral variables. Our approach advances this field in two ways: (1) by leveraging publicly available, longitudinal health datasets to promote inclusivity and scalability, and (2) by combining multiagent orchestration, ontologies, and LLM reasoning to enable principled, explainable feature selection. These contributions distinguish our framework from traditional statistical or machine-learning models and align with emerging calls for DTs that are both interpretable and adaptable to real-world complexity.

It is important to note the distinction between the “simulation-centric” twin presented here and “operational” twins used in acute care. Our system lacks the continuous, real-time data synchronization typical of industrial DTs [1]. Instead, it functions as a progression-aware simulator, a foundational prototype that establishes the semantic and computational architecture (agents, ontologies, and state transitions) required for a DT. While currently powered by static survey waves, this same architecture is designed to ingest real-time streams (eg, wearables) in future iterations.

### Conclusions

This study presents a foundational, progression-aware DT framework for predictive risk projection using public health data, rather than a validated dynamic or clinical DT. We show how large, heterogeneous cohort datasets can be transformed into simulation-capable, longitudinal digital patient representations using ontology-guided feature selection, agent-orchestrated LLM reasoning, and machine learning–based prediction and transition modeling. While the current implementation is offline and limited to 2 survey waves, it establishes a methodological foundation for DT construction as richer and higher-frequency data become available.

A key contribution of this work is demonstrating that public health data, when semantically aligned and processed through an LLM-assisted, agent-orchestrated pipeline, can support not only risk prediction but also predictive simulation. The resulting DT enables the exploration of how model-estimated risk trajectories change under hypothetical modifications to modifiable factors, such as body weight, physical activity, smoking, or psychosocial stress. These simulations reflect model sensitivity rather than real-world intervention effects, but they provide a practical and interpretable way to explore risk dynamics and generate hypotheses in chronic disease research.

For public health planners and clinical researchers, this framework enables population-level “what-if” analyses and supports exploratory individualized risk profiling and scenario analysis grounded in real cohort data. For medical informatics researchers, the proposed architecture, which combines ontologies, LLM-based agents, and transition-aware prediction models, provides a reusable blueprint for other slowly progressing, multidomain conditions, such as CVD, frailty, and COPD.

Future work will focus on the following 4 directions:

1. Extending single-step progression to multistep and higher-frequency updating through integration with EHR, wearable, and mobile-health data;
2. Incorporating decision-analytic and reinforcement-learning components to identify which modifiable factors are most influential for reducing individual risk;
3. Performing external and prospective validation to move from predictive projections toward decision-grade DTs; and
4. Integrating causal-inference and trial-linked data to transition from predictive counterfactuals to causally grounded DT simulation.

## Supplementary material

10.2196/87374 Multimedia Appendix 1 Top 200 features selected by ontology-assisted large language model.

10.2196/87374 Multimedia Appendix 2 Top 200 features selected with Lasso (L1-regularized logistic regression).

10.2196/87374 Multimedia Appendix 3 Top 200 features selected with mutual information.

10.2196/87374 Multimedia Appendix 4 Supplementary analyses of feature selection and latent-state stability.

## Abbreviations

AUC: area under the receiver operating characteristic curve
CVD: cardiovascular disease
DMTO: Diabetes Mellitus Treatment Ontology
DT: digital twin
EHR: electronic health record
FASTO: FHIR And SSN-based Type 1 diabetes Ontology
kNN: k-nearest neighbor
Lasso: L1-regularized logistic regression
LLM: large language model
MI: mutual information
MIDUS: Midlife in the United States
PICO: population, intervention, comparison, and outcome
PR-AUC: precision-recall area under the curve
RAG: retrieval-augmented generation
SHAP: Shapley Additive Explanations

## References

[1] Papachristou K, Katsakiori PF, Papadimitroulas P, Strigari L, Kagadis GC (2024). Digital twins’ advancements and applications in healthcare, towards precision medicine. J Pers Med.

[2] Zhang K, Zhou HY, Baptista-Hon DT (2024). Concepts and applications of digital twins in healthcare and medicine. Patterns (N Y).

[3] Turner K, Hohman KH (2024). Demonstrated progress and future promise of chronic disease data modernization. Prev Chronic Dis.

[4] Dang Y, Li F, Hu X (2023). Systematic design and data-driven evaluation of social determinants of health ontology (SDoHO). J Am Med Inform Assoc.

[5] Wang S, McDermott MBA, Chauhan G, Hughes MC, Naumann T, Ghassemi M MIMIC-extract: a data extraction, preprocessing, and representation pipeline for MIMIC-III.

[6] Orhan F, Kurutkan MN (2025). Predicting total healthcare demand using machine learning: separate and combined analysis of predisposing, enabling, and need factors. BMC Health Serv Res.

[7] Radler BT (2014). The Midlife in the United States (MIDUS) series: a national longitudinal study of health and well-being. Open Health Data.

[8] National Health and Nutrition Examination Survey. Centers for Disease Control and Prevention (CDC).

[9] Health research data for the world. UK Biobank.

[10] Schiavenato M, Chu F (2021). PICO: what it is and what it is not. Nurse Educ Pract.

[11] Pudjihartono N, Fadason T, Kempa-Liehr AW, O’Sullivan JM (2022). A review of feature selection methods for machine learning-based disease risk prediction. Front Bioinform.

[12] Van Hulse J, Khoshgoftaar TM, Napolitano A, Wald R (2012). Threshold-based feature selection techniques for high-dimensional bioinformatics data. Netw Model Anal Health Inform Bioinforma.

[13] Sadeghian Z, Akbari E, Nematzadeh H, Motameni H (2025). A review of feature selection methods based on meta-heuristic algorithms. J Exp Theor Artif Intell.

[14] Gao Y, Xiong Y, Jia K (2023). Retrieval-augmented generation for large language models: a survey. arXiv.

[15] El-Sappagh S, Kwak D, Ali F, Kwak KS (2018). DMTO: a realistic ontology for standard diabetes mellitus treatment. J Biomed Semantics.

[16] Common diabetes medications ontology. NCBO BioPortal.

[17] HL7 FHIR and SSN ontology based type 1 diabetes mellitus ontology. NCBO BioPortal.

[18] BioMedBridges diabetes ontology. NCBO BioPortal.

[19] Study cohort ontology. NCBO BioPortal.

[20] Chari S Making study populations visible through knowledge graphs.

[21] 45 CFR 46.102. Code of Federal Regulations.

[22] Terms of use. ICPSR.

[23] Nohara Y, Matsumoto K, Soejima H, Nakashima N (2022). Explanation of machine learning models using shapley additive explanation and application for real data in hospital. Comput Methods Programs Biomed.

[24] Yang X, Li J (2025). A clustering-based federated deep learning approach for enhancing diabetes management with privacy-preserving edge artificial intelligence. Healthc Anal.

[25] Yang X, Li J Edge AI empowered personalized privacy-preserving glucose prediction with federated deep learning.

[26] Li Q, Amat KR, Li J (2025). LLM-powered personalized glucose prediction in type 1 diabetes. Comput Struct Biotechnol Rep.

