# Supplementary material for: Modeling Diabetes Risk and Progression With Public Health Data: Ontology-Guided, Simulation-Capable Digital Twin Study
Source: JMIR Med Inform. 2026 Apr 21;14:e87374. doi: 10.2196/87374 (PMC13098727; doi:10.2196/87374)
Supplement: Multimedia Appendix 4 [file medinform-v14-e87374-s004.docx]

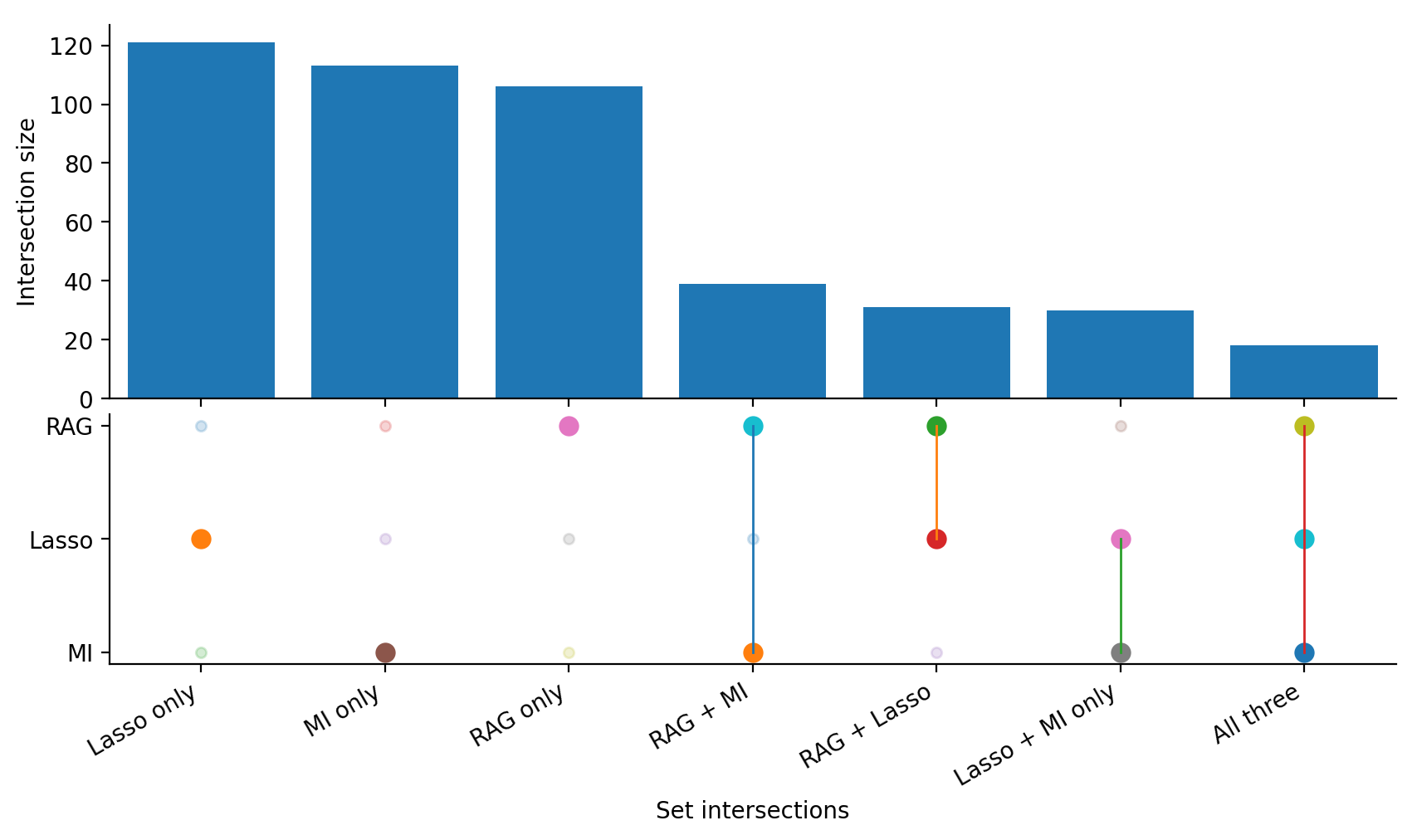


**Figure S1. Overlap of selected feature sets across three selectors (UpSet plot).**

**Table S1. Exact intersection sizes across selectors.**

| **Exact intersection** | **No. of features** |
| --- | --- |
| Lasso only | 121 |
| MI only | 113 |
| RAG only | 106 |
| RAG + MI | 39 |
| RAG + Lasso | 31 |
| Lasso + MI only | 30 |
| All three | 18 |

**Table S2. Pairwise overlap and Jaccard similarity between feature sets.**

| **Comparison** | **Overlap** | **Jaccard** |
| --- | --- | --- |
| RAG vs Lasso | 49 | 0.142 |
| RAG vs MI | 57 | 0.169 |
| Lasso vs MI | 48 | 0.136 |
| All three | 18 |  |
| Union size | 458 |  |

**Table S3. Construct composition proxy by MIDUS module (counts in Top-200).**

| **Module** | **RAG** | **Lasso** | **MI** |
| --- | --- | --- | --- |
| Physical activity (any) | 21 | 4 | 6 |
| Weight management | 5 | 0 | 5 |
| Prescription medicine used | 7 | 31 | 8 |
| Body measurement | 5 | 2 | 6 |
| Activity of daily living | 4 | 8 | 11 |
| Resources for obtaining healthcare | 10 | 9 | 4 |
| Self characteristics | 6 | 0 | 0 |
| Family support/strain | 12 | 5 | 4 |
| Stop-and-go switch task | 22 | 33 | 23 |
| Immediate word list recall | 9 | 3 | 4 |

**Table S4.  Ablation Study – Feature Selection Method Comparison**

| **Feature Selection Method** | **# Features Selected** | **AUC (Prediction)** | **"Noise" Features Included*** | **Clinical Relevance Score**** |
| --- | --- | --- | --- | --- |
| Baseline 1: Mutual Information | 200 | 0.78 | Medium (110) | Low |
| Baseline 2: LASSO (L1) | 200 | 0.73 | High (135) | Medium |
| Ours (Ontology + LLM Agent) | 200 | 0.82 | Low(70) | High |

**Table S5. Semantic-audit category counts (derived from Figure 3).**

| **Semantic category** | **RAG-Agent (ours)** | **Mutual information** | **Lasso** |
| --- | --- | --- | --- |
| Psychosocial determinants | 15 | 7 | 10 |
| Core metabolic risk factors | 13 | 10 | 6 |
| Longitudinal trajectories | 3 | 4 | 2 |
| Administrative artifacts | 0 | 0 | 3 |
| Spurious biological correlates | 1 | 0 | 9 |
| Micro-level experimental noise | 22 | 23 | 33 |

**Table S6a. RAG-only exemplar features (Top-10).**

| **Feature ID** | **Item name** | **MIDUS module/path** | **Selection score** |
| --- | --- | --- | --- |
| B1SE15 | Age would like to be | Survey > Personal Beliefs > Self Characteristics | 0.800 |
| B1SA6D | Compare weight now to 5 years ago | Survey > Your Health > Comparative Health/Memory Items | 0.800 |
| B1PB1 | Respondent education level | Survey > Education, Occupation, and Marital Status > History of Marriage | 0.800 |
| B1SE2J | Physical fitness important for good life | Survey > Personal Beliefs > EDWB Categories for Living a Good Life | 0.800 |
| B1SA30C | Vigorous chores activity frequency - Summer | Survey > Your Health > Vigorous Physical Activity | 0.700 |
| B1SA31F | Moderate leisure activity frequency - Winter | Survey > Your Health > Moderate Physical Activity | 0.700 |
| B1SA30F | Vigorous leisure activity frequency - Winter | Survey > Your Health > Vigorous Physical Activity | 0.700 |
| B1SA30A | Vigorous job activity frequency - Summer | Survey > Your Health > Vigorous Physical Activity | 0.700 |
| B1SA30B | Vigorous job activity frequency - Winter | Survey > Your Health > Vigorous Physical Activity | 0.700 |
| B1SA43B | Lose 10 pounds by diet or exercise | Survey > Your Health > Weight Management | 0.700 |

**Table S6b. Lasso-only exemplar features (Top-10).**

| **Feature ID** | **Item name** | **MIDUS module/path** | **Selection score** |
| --- | --- | --- | --- |
| B1SA11P | Lupus or autoimmune disorder ever - 12 months | Survey > Your Health > Prescription Medicine Used | 0.443 |
| B1SA11N | Varicose veins ever - 12 months | Survey > Your Health > Prescription Medicine Used | 0.398 |
| B1SA11C | Other lung problems ever - 12 months | Survey > Your Health > Prescription Medicine Used | 0.396 |
| B1SA11DD | Swallowing problems ever - 12 months | Survey > Your Health > Prescription Medicine Used | 0.387 |
| B1SA11L | Gall bladder trouble ever - 12 months | Survey > Your Health > Prescription Medicine Used | 0.384 |
| B1SA11CC | Piles or hemorrhoids ever - 12 months | Survey > Your Health > Prescription Medicine Used | 0.379 |
| B1SA11BB | Hernia ever - 12 months | Survey > Your Health > Prescription Medicine Used | 0.366 |
| B1SA11K | Constipated all or most ever - 12 months | Survey > Your Health > Prescription Medicine Used | 0.365 |
| B1SA11Q | Gum or mouth trouble persistent ever - 12 months | Survey > Your Health > Prescription Medicine Used | 0.361 |
| B1SA11U | Alcohol or drug problem ever - 12 months | Survey > Your Health > Prescription Medicine Used | 0.358 |

**Table S6c. MI-only exemplar features (Top-10).**

| **Feature ID** | **Item name** | **MIDUS module/path** | **Selection score** |
| --- | --- | --- | --- |
| B1SA7A | Compare overall health to others your age | Survey > Your Health > Comparative Health/Memory Items | 0.023 |
| B1SA28H | Health limits walking one block | Survey > Your Health > Activity of Daily Living | 0.017 |
| B1SA28B | Health limits bathing or dressing self | Survey > Your Health > Activity of Daily Living | 0.015 |
| B1PBYEAR | Birth year at interview | Survey > Administration > Respondent Birth Year | 0.015 |
| B1SI4 | Years lived in this state | Survey > Your Neighborhood > Years Lived in | 0.015 |
| B1SE7V | Fun learning to walk tightrope | Survey > Personal Beliefs > Multidimensional Personality | 0.015 |
| B1SE10D | Hardly ever expect things to go my way | Survey > Personal Beliefs > Life Event Checklist | 0.015 |
| B1SP2F | People think you are dishonest - frequency | Survey > Discrimination > Daily Discrimination (Frequency) | 0.015 |
| B1SI6B | Safe alone neighborhood at night | Survey > Your Neighborhood > Feelings about Neighborhood/Home | 0.014 |
| B1SA53A | Number times physical routine exam - 12 months | Survey > Your Health > Physical Health Care Utilization | 0.014 |

**Table S7. Stability Analysis of Latent States (K = 3–5)**

| Configuration | "High Risk" Cluster Size (N) | | Mean HbA1c of Cluster | Jaccard Overlap w/ K=3 Baseline* | Interpretation of New Clusters |
| --- | --- | --- | --- | --- | --- |
| K = 3 (Selected) | | 142 | 7.4% | 1.00 (Ref) | Distinct: Healthy, Pre-Diabetic, Diabetic |
| K = 4 | | 138 | 7.5% | 0.96 | "Healthy" splits into "Optimal" vs "Normal"; "Diabetic" stays stable. |
| K = 5 | | 135 | 7.6% | 0.91 | "Pre-Diabetic" splits into "Early Risk" vs "High Risk". |
